# An Approach to Improve the Performance of PM Forecasters

**DOI:** 10.1371/journal.pone.0138507

**Published:** 2015-09-28

**Authors:** Paulo S. G. de Mattos Neto, George D. C. Cavalcanti, Francisco Madeiro, Tiago A. E. Ferreira

**Affiliations:** 1 Centro de Informática, Universidade Federal de Pernambuco, Recife, Pernambuco, Brazil; 2 Universidade Católica de Pernambuco, Recife, Pernambuco, Brazil; 3 Departamento de Estatística e Informática, Universidade Federal Rural de Pernambuco, Recife, Pernambuco, Brazil; Universidad Veracruzana, MEXICO

## Abstract

The particulate matter (PM) concentration has been one of the most relevant environmental concerns in recent decades due to its prejudicial effects on living beings and the earth’s atmosphere. High PM concentration affects the human health in several ways leading to short and long term diseases. Thus, forecasting systems have been developed to support decisions of the organizations and governments to alert the population. Forecasting systems based on Artificial Neural Networks (ANNs) have been highlighted in the literature due to their performances. In general, three ANN-based approaches have been found for this task: ANN trained via learning algorithms, hybrid systems that combine search algorithms with ANNs, and hybrid systems that combine ANN with other forecasters. Independent of the approach, it is common to suppose that the residuals (error series), obtained from the difference between actual series and forecasting, have a white noise behavior. However, it is possible that this assumption is infringed due to: misspecification of the forecasting model, complexity of the time series or temporal patterns of the phenomenon not captured by the forecaster. This paper proposes an approach to improve the performance of PM forecasters from residuals modeling. The approach analyzes the remaining residuals recursively in search of temporal patterns. At each iteration, if there are temporal patterns in the residuals, the approach generates the forecasting of the residuals in order to improve the forecasting of the PM time series. The proposed approach can be used with either only one forecaster or by combining two or more forecasting models. In this study, the approach is used to improve the performance of a hybrid system (HS) composed by genetic algorithm (GA) and ANN from residuals modeling performed by two methods, namely, ANN and own hybrid system. Experiments were performed for PM_2.5_ and PM_10_ concentration series in Kallio and Vallila stations in Helsinki and evaluated from six metrics. Experimental results show that the proposed approach improves the accuracy of the forecasting method in terms of fitness function for all cases, when compared with the method without correction. The correction via HS obtained a superior performance, reaching the best results in terms of fitness function and in five out of six metrics. These results also were found when a sensitivity analysis was performed varying the proportions of the sets of training, validation and test. The proposed approach reached consistent results when compared with the forecasting method without correction, showing that it can be an interesting tool for correction of PM forecasters.

## Introduction

Air pollution has been the focus of public concern due to its health impact on the worldwide population, mainly in the big urban centers [1, 2]. The contamination of the earth’s atmosphere by biological molecules, particulates and other harmful substances causes diseases and death in humans, who are also harmed by the damage that other living organisms, such as food crops, natural vegetation and herds of animals, suffer [2].

Particulate matter (PM) concentration has been a major concern among the air pollutants as according to epidemiological studies [3–12] and several diseases have been associated with this substance [1]. The Global Monitoring Report [1] points out PM as the major urban air pollutant affecting human health. The level of damage usually depends up on the duration of exposure as well as the kind and concentration of particles in the air [2, 4, 7, 9]. In general, the short-term effects [1, 13, 14], such as irritation in the eyes, nose and throat, headaches, nausea and allergic reactions are less serious [3]. However, in some cases, the exposure to short-term air pollution can cause upper respiratory infections such as bronchitis and pneumonia and aggravate the medical conditions of individuals with asthma and emphysema [3]. The long term effects [1, 8] may include chronic respiratory disease [3], lung cancer [7], cardiovascular diseases [5], such as ischemia-reperfusion injury and atherosclerosis, and even damage to the brain [15, 16], [15, 16], liver [16, 17], or kidneys [17, 18]. Continuous exposure to air pollution [8, 9] can severely affect the health and growth of children and may aggravate medical conditions in the elderly.

The monitoring of PM concentration is a relevant issue, as it allows the governments to create public policies to prevent and warn the population regarding high levels of PM. In this scenario, Artificial Neural Networks (ANN) have been widely used for the forecasting of PM concentration [19]. A non-exhaustive search in the literature points out three general ANN-based approaches for forecasting of PM concentration: the use of an ANN itself, hybrid systems that use search algorithms for the choice of ANN parameters, and hybrid systems that combine an ANN with another forecaster. Several studies belonging to each one of the aforementioned approaches are addressed in the following.

Four different ANN models: Recurrent Network Model (RNM), Change Point Detection Model with RNM, Sequential Network Construction Model and Self Organizing Feature Model were considered by Sharma *et al.* [20] for forecasting the concentration data of seven pollutants, among them PM_2.5_ and PM_10_, in the California area. A Multilayer Perceptron (MLP) model, a Radial Basis Function (RBF) and a Square Multilayer Perceptron (SMLP) were addressed by Ordieres *et al.* [21] for forecasting PM_2.5_ concentration in the cities of El Paso (Texas) and Ciudad Juárez (Chihuahua). Other studies also used an MLP model: Kukkonen *et al.* [22] used an MLP model with homoscedastic and heteroscedastic Gaussian noise (ANN-HeG) to forecast the PM_10_ concentration in Helsinki, Caselli *et al.* [23] compared an MLP training via backpropagation, an RBF model and a multivariate regression model to forecast daily PM_10_ in Bari, Italy, and Gennaro *et al.* [24] used the MLP model developed in [23] with a specified set of input data to forecast PM_10_ concentration in two sites in the Western Mediterranean. Forecasting the maximum average concentration of PM_10_ per day in the city of Santiago, Chile, was carried out by Perez and Reyes [25] with the use of ANN. The majority of studies concerning PM concentration forecasting are regarded with one-step ahead forecasting. Multi-step ahead forecasting is also found in the literature. For example, Kurt and Oktay [26] used an MLP model to forecast sulfur dioxide (SO_2_), carbon monoxide (CO) and PM_10_ concentration levels for 3 days ahead for a Besiktas district (Istanbul, Turkey) and Caselli *et al.* [23] applied an MLP model to forecast PM_10_ concentrations for 1, 2 and 3 days ahead.

Intelligent hybrid systems have also been proposed through combinations of ANNs with other techniques. These techniques are generally employed for the selection of input variables and the best ANN parameters, such as number of neurons in hidden and input layers, activation function, and training algorithm among others. Examples of techniques that have been combined with ANN include: Principal Component Analysis (PCA) [27], which was used with MLP to forecast PM_10_ concentration in Thessaloniki and for selection of input variables, followed by forecasting of PM_2.5_ and PM_10_ in Thessaloniki and Helsinki via MLP and linear regression (LR) [28]; Genetic Algorithm (GA), which was applied to select the inputs for ANN to forecast PM_10_ emission in 26 Europe countries [29] and a Multi-Objective Genetic Algorithm (MOGA), which was applied to reduce the number of potential meteorological input variables, with the integration of an MLP model with a numerical weather prediction model HIRLAM (High Resolution Limited Area Model) to forecast sequential hourly time series concentrations of PM_2.5_ in Helsinki [30]; Nearest Neighbor method combined with a MLP model in the scenario of PM_10_ concentration in Santiago [31]; Wavelets with combination of an ANN ensemble to forecast the daily average concentration of PM_10_ in Warsaw, Poland [32]. Hybrid systems were also proposed in other studies: Mishra *et al.* [33] proposed a Neuro-fuzzy model for forecasting PM_2.5_ during haze conditions in Delhi, India, and Qin *et al.* [34] proposed a hybrid model based on Cuckoo search (CS) and ANN training via backpropagation to forecast PM concentration levels in the four major cities of China (Beijing, Shanghai, Guangzhou and Lanzhou). Hybrid systems are also employed for multi step ahead forecasting. For example, Ul-Saufie *et al.* [35] combined MLR and ANN with PCA to forecast daily PM_10_ concentration (one, two and three days ahead) in Negeri Sembilan, Malaysia.

Hybrid systems have been proposed assuming that one forecaster can be insufficient to model a time series [36–38], making the combination of two or more models necessary. In this context, two assumptions are adopted: (i) a time series can contain patterns that are not purely linear or nonlinear [36]; (ii) a highly nonlinear time series cannot be modeled by an ANN alone [37, 38]. In the first assumption, the use of only linear or non-linear techniques may lead to inaccurate results, making it necessary to develop models considering both the linearity and the non-linearity involved in the time series [36]. In the second assumption, the use of one ANN can be insufficient due to problems of misspecification, generating a biased or inconsistent model [37, 38]. The misspecified ANN could be generated either during structure selection or during training, causing overfitting or underfitting problems.

A particular class of hybrid systems, which uses multiforecasters, explores the error series (residuals) in an attempt to improve the forecasting. Given the forecasting of a model, the error series (residuals) is obtained from the difference between actual time series and its forecasting. Usually, it is supposed that the error series is a white noise, i.e., it consists of independent and identically distributed random shocks that are unpredictable [37, 38]. However, due to misspecification of the forecaster, or time series behavior (linear and non-linear components), or due to disturbances present in the stochastic process after the specification of the forecaster, the assumption of the white noise can be violated. Thus, the temporal patterns that still remain in the error series can be captured and used to generate the forecasting of the residuals [37, 38].

In the scenario of air pollutant concentration forecasting, hybrid systems with more than one forecaster and using ANN have been proposed. Al-Alawi *et al.* [39] proposed a hybrid method using multiple regression combined with both PCA and ANN to forecast the concentration of ozone in Kuwait’s lower atmosphere. Ettouney *et al.* [40] also used a PCA combined with two ANNs in cascade to forecast ozone concentration in two locations in Kuwait. Westerlund *et al.* [41] proposed a forecast combination (FC), using two methods: linear regression and ANN to forecast air quality in Bogota. Combinations with Autoregressive Integrated Moving Average (ARIMA) model were also proposed: Sánchez *et al.* [42] combined an Elman neural network with ARIMA for forecasting of SO_2_ concentration registered in a control station in the vicinity of a coal-fired power station in northern Spain and Díaz-Robles *et al.* [43] proposed a hybrid ARIMA and ANN model to forecast PM_10_ concentration levels in urban areas of Temuco, Chile.

Hybrid models have not been applied only for forecasting parameters regarding air quality. In the literature, one can observe hybrid models in other public health interest scenarios such as: ARIMA combined with generalized regression neural network (GRNN) to forecast the incidence of tuberculosis [44], ARIMA combined with nonlinear auto-regressive neural network (NARNN) to forecast incidence of hand-foot-mouth disease (HFMD) [45] and ARIMA combined with NARNN to forecast the prevalence of schistosomiasis in humans [46].

**Fig 1:**
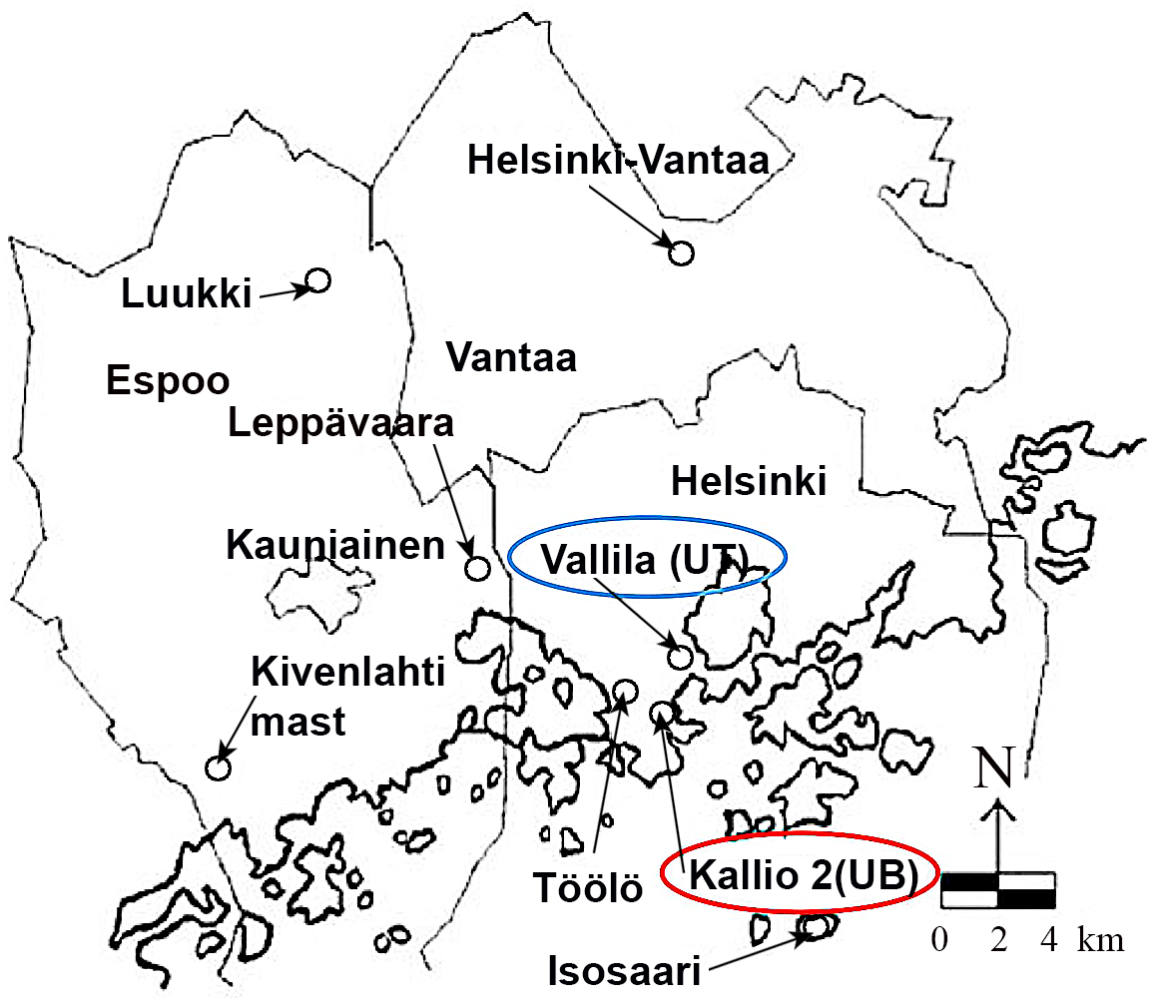
Locations of the Kallio and Vallila air quality stations in Helsinki. The Vallila station is located in urban traffic (UT) environment and the Kallio station is located in urban background (UB) environment(adapted from [47]).

Herein, we propose a hybrid approach for improving the performance of PM concentration forecasters using residual modeling. The hybrid approach consists of a recursive modeling, which at each iteration verifies if there are temporal patterns in the remaining residuals, thus aiming to generate the forecasting of the error series. Then, the forecasting of the error series is used in the next stage of the sequence of forecasters in order to improve the forecasting. This proposed approach differs from previous multiforecaster hybrid systems based on error series in the fact that those ones use only one residual series. The proposed hybrid system uses as many residual series as necessary to obtain a residual with white noise behavior. Another highlight of the proposed approach is its generality, that is, the forecasting system is sufficiently versatile to use a set of different forecasters, e.g. an ANN followed by an ARIMA model, or an ARIMA model followed by an ANN, or a sequence of three different forecasters. Simulation results are presented for four time series: PM_2.5_ and PM_10_ daily concentration levels in Kallio and Vallila stations in Helsinki, Finland shown in Fig 1. More specifically, in the present study, error series are used to improve the forecasting of a recent hybrid system composed of genetic algorithm (GA) and ANN presented in the literature [19]. For residual modeling, two methods are considered in this study: an MLP neural network and our own hybrid system used in PM concentration series forecasting. The results are evaluated using a set of six well-known metrics and show that the proposed approach is capable of improving the performance of the PM forecaster considered for all cases.

## The Methodology for the Correction of Pollutant Forecasters

**Fig 2:**
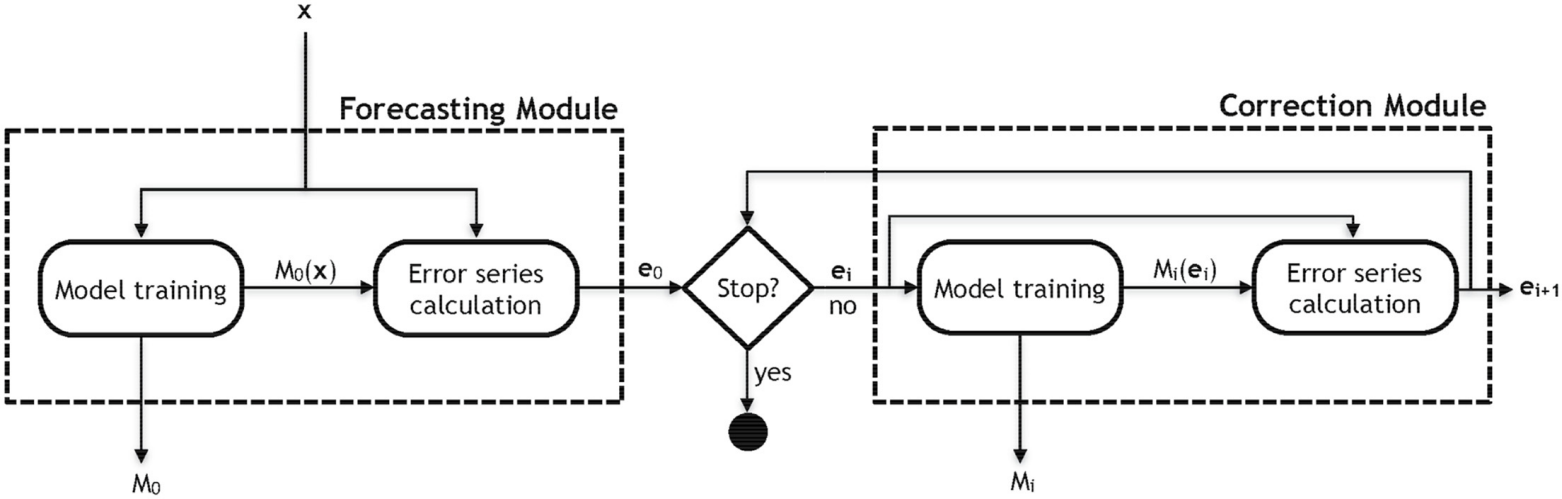
Training architecture of the proposed system.

Fig 2 shows the architecture of the proposed approach, which is composed of two modules: (i) Forecasting and (ii) Correction. Given a univariate time series (**x**), the aim of the first module is to train a forecasting model (*M*_0_). Then, the error series **e**_0_ is calculated as the difference between the time series and the output of the model, **e**_0_ = **x** − *M*_0_(**x**).

The Correction Module is only executed if the residual series (**e**_0_) is not a white noise. In other words, a test (Fig 2—“Stop?”) is performed to verify if there is enough information in the error series to improve the precision of the forecasting.

Formally, a white noise [48] is a sequence of independent and identically distributed random values with zero mean and constant variance. In general, the literature of time series supposes that the residuals generated by the forecasters have a white noise behavior, and the module (i) Forecasting of the proposed approach (Fig 2) shows this classical approach. However, we expect that the error series contains useful information that was not captured by the forecaster *M*_0_ (module (i) Forecasting). In this context, there are tests [48] to check the hypothesis that the residuals are independent and identically distributed random variables. Some of these tests are: the *portmanteau* test, spectral analysis, turning point test and autocorrelation function (ACF) [48]. The ACF test is adopted in this study and consists of the cross-correlation of a series with itself at different times, as a function of two time lags. So, ACF measures the correlation between the value of a series in the time *t* and *t* + *k* according to Eq (1):

$$\begin{array}{c} \rho_{k}=\text{Corr}\left(y_{t},y_{t+k}\right)=\frac{\gamma_{h}}{\gamma_{0}}, \end{array}$$ (1)

where Corr is the correlation, *γ*_*h*_ = Cov(*y*_*t*_, *y*_*t*+*k*_), where Cov is the covariance, and *γ*_0_ is the sample variance of the time series. *ρ*_*k*_ lies in the range [−1, 1], where 1 and −1 indicate perfect correlation and perfect anti-correlation, respectively. If the values of *ρ*_*k*_, where *k* = 1, …, *n*, are in the range [−2*s*, 2*s*] (where *s* is the standard deviation of data sample), then there is no correlation; otherwise, there is correlation in the series data.

Based on the assumption that it is possible to extract valuable information from the error series (**e**_0_) to improve the forecasting of the whole system, the Correction Module trains a model (*M*_1_) that aims to forecast the error of the model (*M*_0_). Once again, if the residual series (**e**_1_), where **e**_1_ = **e**_0_ − *M*_1_(**e**_0_), of the model **M**_1_ is not a white noise, this procedure is repeated until the stop criterion is reached. So, the output of the Correction Module is given by two sets: models {*M*_0_, *M*_1_, …, *M*_*n*_} and error series {**e**_0_, **e**_1_, …,**e**_*n*−1_}.

**Fig 3:**
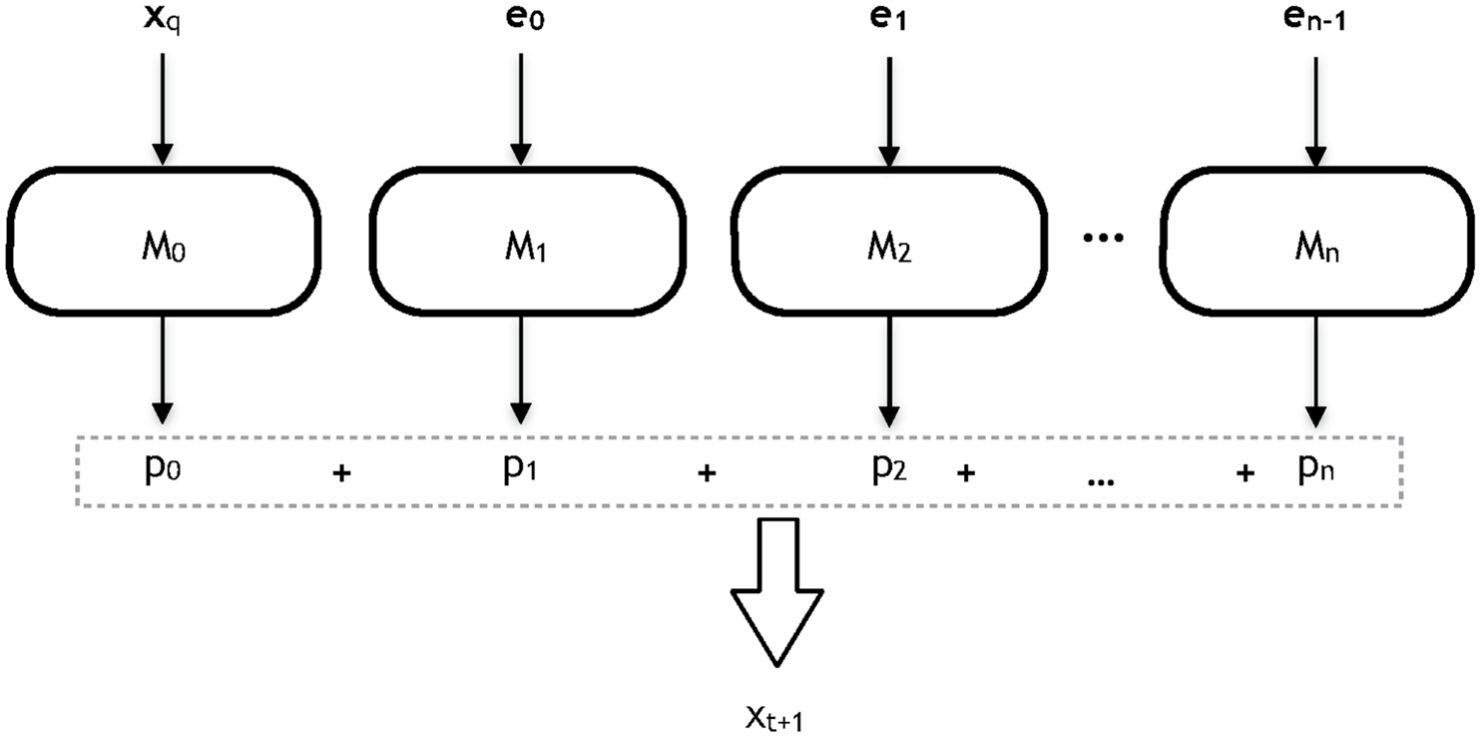
Test architecture of the proposed system.

After training, the models are used to forecast unseen patterns as shown in Fig 3. So, given a time series **x**_*q*_ = [*x*_1_, *x*_2_, …, *x*_*t*_], we want to forecast *x*_*t*+1_. The predicted value of *x*_*t*+1_ is given by Eq (2).

$$\begin{array}{c} {\hat{x}}_{t+1}=M_{0}\left(x_{q}\right)+M_{1}\left(e_{0}\right)+M_{2}\left(e_{1}\right)+\ldots +M_{n}\left(e_{n-1}\right), \end{array}$$ (2)

where ideally it is expected that the contribution of the *M*_*i*−1_ model is greater than that of the *M*_*i*_ model to the final forecasting ${\hat{x}}_{t+1}$ of the proposed approach. This should occur assuming that if the remaining error series is not a white noise, at each iteration *i* of the proposed approach a model *M*_*i*_ captures temporal patterns. In this context, it is expected that the contributions of the *M*_*i*_ models decrease at each iteration, remaining only a signal uncorrelated in time; in other words, a white noise.

## Simulation and Results

Four time series were used for the evaluation of the proposed approach. The series correspond to daily mean concentration of particulate matter (PM_2.5_ and PM_10_) from Helsinki. The data set is composed of values measured between the years 2001 to 2003 from stations Kallio and Vallila [28]. Despite the two stations be located in Helsinki, they have different characteristics. The Kallio station is located in an urban background and the Vallila station is situated in an area more exposed to pollution from local traffic.

The data set is normalized to lie within the interval [0, 1] and divided into three sets: 80% for training, 10% for validation and 10% for test. For each time series, ten simulations with the proposed approach were performed and the best model was selected based on the performance (fitness value) in the validation data set. The results for all models correspond to the one step ahead forecasting from the test set. Table 1 shows the six metrics [28, 49, 50] used to evaluate the performance of the approach employed in this paper: Mean Squared Error (MSE), Mean Absolute Percentage Error (MAPE), U of Theil Statistics (U), Average Relative Variance (ARV), Prediction of Change in Direction (POCID) and Index of Agreement (IA). For MSE, MAPE, U and ARV, the lower the value of those measures, the better is the forecasting of the model. U and ARV measures are used to compare the model performances with the forecasting of a Random Walk model and that of the mean of the series, respectively. If U and ARV values are equal to 1, the forecasting of the forecaster is equivalent to the random walk model (U = 1), or to the mean of the time series (ARV = 1), respectively. However, if U and ARV values are less than or greater than 1, the forecasting of the model is better or worse than the performance of the random walk model or mean, respectively. In case of POCID and IA, the higher the value the better is the performance of the model. The POCID can have values in the range [0, 100] and IA in the range [0, 1]. A ratio shown in Eq (3) was developed to compare the performances of the current correction with the model added and the previous correction, or the model without correction:

$$\begin{array}{c} ratio=\frac{EvaluationMeasure_{correctedModel}}{EvaluationMeasure_{uncorrectedModel}}, \end{array}$$ (3)

where *EvaluationMeasure*_*correctedModel*_ is the forecaster’s performance reached after the current correction (*n*) is added and *EvaluationMeasure*_*uncorrectedModel*_ is the value of forecaster’s performance reached with the previous correction (*n* − 1, model without the current correction). For POCID, IA and fitness values, if the obtained value in Eq (3) is greater than 1, the correction improves the forecasting. If the value is equal to or less than 1, the correction either does not add information, or worsens the previous forecasting, respectively.

**Table 1:** Metrics for forecasting assessment, where *N* is the size of the series, target_*j*_ is the real value at period *j*, output_*j*_ is the forecasting at period *j* and $\bar{\text{target}}$ is the mean of the series. In the column *θ*: ↑ means the higher the value of metric the better is the forecasting, and ↓ means the lower the value of metric the better is the forecasting.

Metric	Equation	Range	*θ*
Mean Squared Error (MSE)	$\text{MSE}=\frac{1}{N}\sum_{j=1}^{N}{\left({\text{target}}_{j}-{\text{output}}_{j}\right)}^{2}$	[0, +∞)	↓
Mean Absolute Percentage Error (MAPE)	$\text{MAPE}=\frac{100}{N}\sum_{j=1}^{N}|\frac{{\text{target}}_{j}-{\text{output}}_{j}}{{\text{target}}_{j}}|$	[0, +∞)	↓
U of Theil Statistics (U)	$\text{U}=\frac{\sum_{j=1}^{N}{\left({\text{target}}_{j}-{\text{output}}_{j}\right)}^{2}}{\sum_{j=1}^{N}{\left({\text{output}}_{j}-{\text{output}}_{j+1}\right)}^{2}}$	[0, +∞)	↓
Average Relative Variance (ARV)	$\text{ARV}=\frac{\sum_{j=1}^{N}{\left({\text{output}}_{j}-{\text{target}}_{j}\right)}^{2}}{\sum_{j=1}^{N}{\left({\text{output}}_{j}-\bar{\text{target}}\right)}^{2}}$	[0, +∞)	↓
Prediction of Change in Direction (POCID)	$\text{POCID}=100\frac{\sum_{j=1}^{N}D_{j}}{N}$, $D_{j}=\{\begin{array}{cc} 1, & \text{if}\;\left({\text{target}}_{j}-{\text{target}}_{j-1}\right)\left({\text{output}}_{j}-{\text{output}}_{j-1}\right)>0.\\ 0, & \text{otherwise}. \end{array}$	[0, 100]	↑
Index of Agreement (IA)	$\text{IA}=1-\frac{\sum_{j=1}^{N}{\left|{\text{output}}_{j}-{\text{target}}_{j}\right|}^{2}}{\sum_{j=1}^{N}{\left(|{\text{output}}_{j}-\bar{\text{target}}|+|{\text{target}}_{j}-\bar{\text{target}}|\right)}^{2}}$	[0, 1]	↑

The proposed approach is used to correct a hybrid system (model of the Forecasting Module—Fig 2) composed by GA and ANN of type MLP [19]. This hybrid system consists of two stages: optimization of the ANN parameters performed by GA and phase adjustment. In the first stage, the GA searches the best configuration of the following parameters of MLP: number of input neurons (relevant time lags), number of nodes in the hidden layer and the training algorithm among four candidates (Levenberg-Marquardt, Scaled Conjugated Gradient, Resilient Backpropagation (RPROP) and One Step Secant Conjugate Gradient).

The search process performed by GA is guided by fitness function, defined in Eq (4). Silva *et al.* [51] reported that the definition of an adequate fitness function is a non-trivial task. So, the used fitness function aims to aggregate different measures and the higher (as closer to 100) its value the more accurate is the forecasting model.

$$\begin{array}{c} Fitness=\frac{POCID}{1+MSE+MAPE+U+ARV}. \end{array}$$ (4)

In the second stage, if necessary, a procedure of phase adjustment is performed aiming to minimize the difference between the forecasting performed by ANN and the PM concentration series. The hybrid system [19] was chosen because it reached superior results in terms of accuracy, when compared with literature [19, 50, 52]. Thus, if the proposed approach is capable of improving a forecaster with high accuracy, it is expected that the approach also improves the forecaster with lower accuracy.

Two models are adopted for correction (Correction Module—Fig 2): the own hybrid system and an MLP model. In both cases, the models are trained recursively until a stopping condition is reached. For ANN, the input and hidden nodes are established using ACF and trial and error method in the interval [1, 20], respectively.

The parameters used in this work are described below:

- The stopping conditions for recursive approach are: (i) the error series has a white noise behavior, or; (ii) an increase (≥ 5%) in the MAPE value with the addition of one correction;
- The parameters of the hybrid system are set up to: (i) Initial acceptable fitness value (1% of error); (ii) Initial maximum number of time lags (10); (iii) Maximum number of hidden units (20); (iv) Maximum number of iterations (10);
- The parameters of the GA used by the hybrid system are set up to: (i) Mutation probability of 10%; (ii) Population size of 10 individuals; (iii) Maximum number of generations equals 1000; (iv) Minimum fitness progress of 10^−4^. Three stopping conditions for the ANN training algorithms are used: (i) Maximum number of iterations equals 1000; (ii) cross-validation process with a generation loss of 5%; (iii) Progress training of 10^−6^.
- The parameters of the ANN of type multi-layer perceptron (MLP) used in the correction phase are: (i) training algorithm is Levenberg-Marquardt; (ii) Maximum number of iterations. Three stopping conditions for Levenberg-Marquardt algorithm are used: (i) Maximum number of iterations equals 1000; (ii) cross-validation process with generation loss of 5%; (iii) Progress training of 10^−6^.

### Correction via Artificial Neural Network

Table 2 shows the results in terms of the evaluation measures and fitness reached by the hybrid system without correction and proposed methodology using ANN for PM_2.5_ and PM_10_ time series from Kallio and Vallila stations. The hybrid system uncorrected (without correction) is named HS. The version of the hybrid system corrected, denoted by HS+C_*n*_, corresponds to the proposed methodology, with *n* terms of corrections generated by an ANN model. For all time series analyzed here there was the need for just one correction term, suggesting that the HS was not capable of capturing all information contained in the time series. Therefore, a comparison is done between HS and HS+C_1_. After the first correction (HS+C_1_), the same stopping criterion was reached for all cases, which was the model residual for a white noise.

**Table 2:** Results with correction via ANN for all series. The best result for each metric and fitness is highlighted in bold.

Station	Series	Models	MSE	POCID	U	MAPE	ARV	IA	Fitness
**Kallio**	PM_10_	HS	6.00E-04	**97.16**	0.0969	35.93	0.3077	0.950	2.60
		HS+C_1_	**5.79E-04**	87.04	**0.0847**	**31.40**	**0.1961**	**0.963**	**2.66**
	PM_2.5_	HS	3.00E-04	**97.19**	0.0596	27.41	0.1349	0.970	3.40
		HS+C_1_	**2.95E-04**	87.74	**0.0510**	**19.18**	**0.0888**	**0.981**	**4.32**
**Vallila**	PM_10_	HS	3.00E-04	**97.82**	**0.0727**	29.66	0.1910	0.960	3.16
		HS+C_1_	3.00E-04	88.57	0.0785	**25.06**	**0.1864**	**0.964**	**3.36**
	PM_2.5_	HS	**1.00E-04**	**98.11**	**0.0313**	21.66	**0.0630**	0.980	4.31
		HS+C_1_	2.01E-04	94.57	0.0450	**15.27**	0.0850	**0.990**	**5.76**

Table 2 shows that the proposed approach improved 15 out of 24 evaluation measures, achieving the best fitness for all time series. The best result for each metric and fitness is highlighted in bold. For pollutant concentration time series from Kallio station, the proposed approach improved almost all evaluation metrics, except the POCID measure. The decrease in MSE and MAPE shows that the correction was able to improve the forecasting of the uncorrected model. For Vallila station, the improvement occurred in MAPE, ARV and IA for PM_10_ concentration time series and in MAPE and IA for PM_2.5_ concentration time series. These results show that for all studied cases, HS+C_1_ reached a better performance than HS with respect to the fitness function, that is the objective for the proposed method, and, as a consequence, it was also observed an improvement in the performance for other metrics.

**Fig 4:**
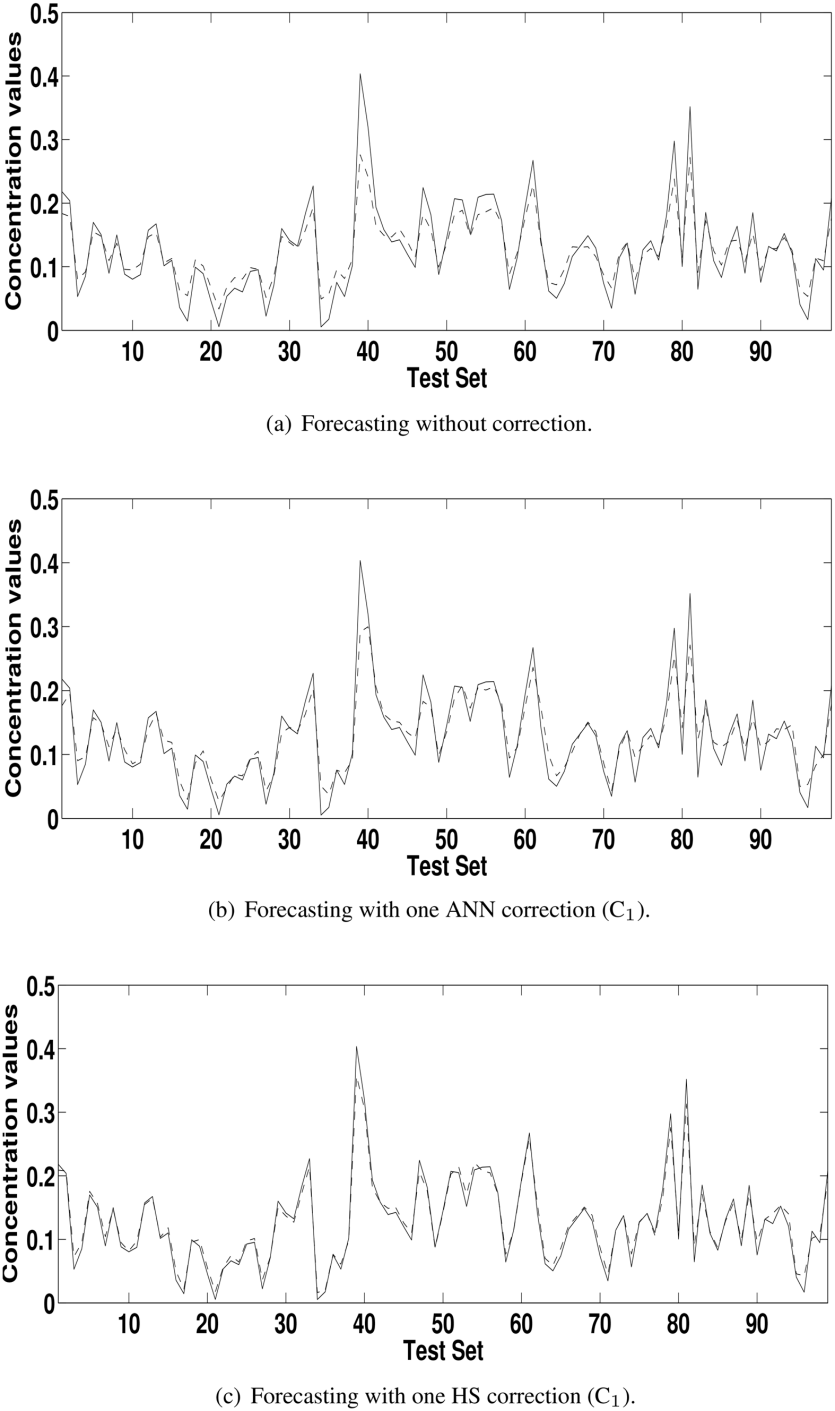
Forecasting for the PM_10_ concentration time series for Kallio Station (solid lines—actual values; dashed lines—predicted values).

**Fig 5:**
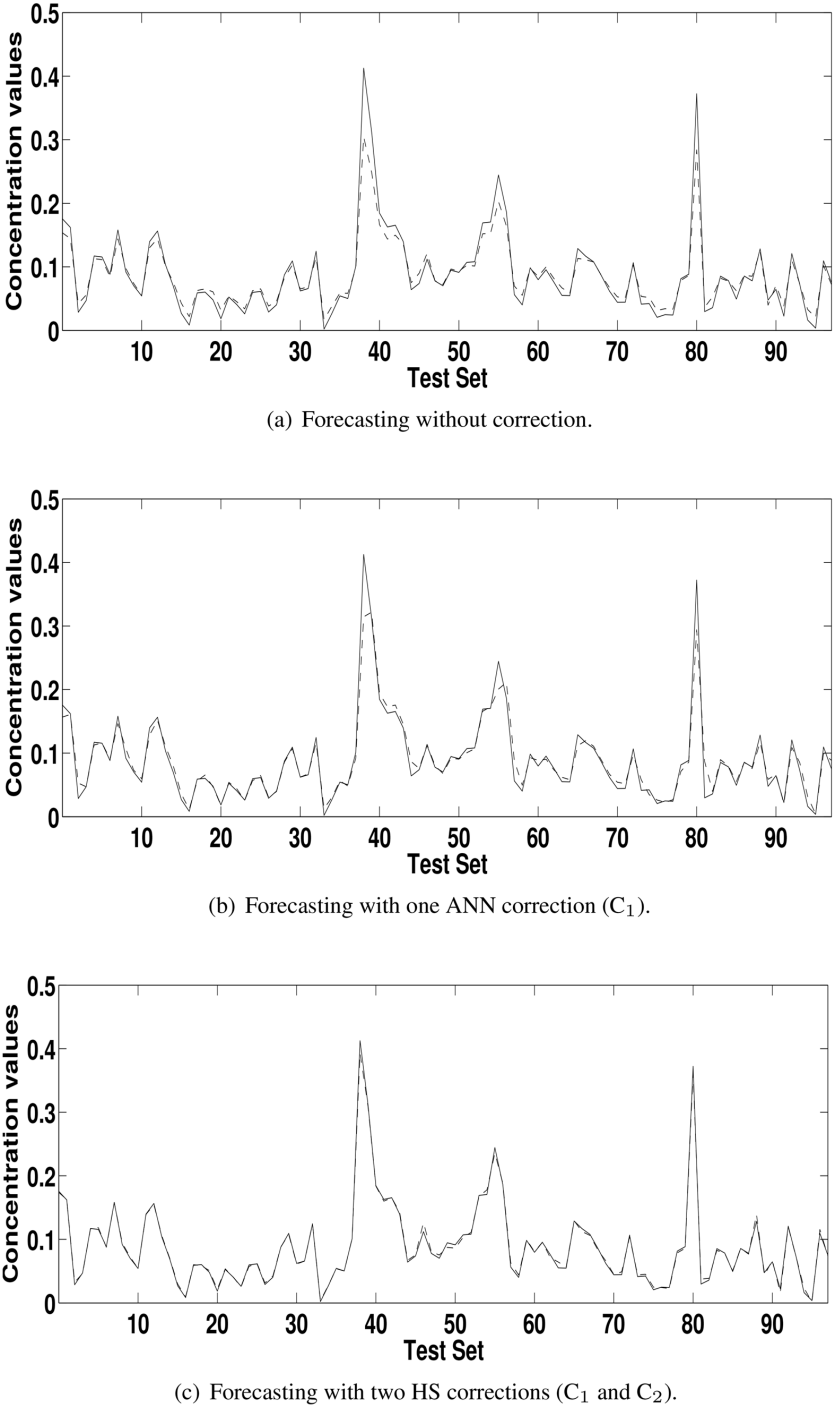
Forecasting for the PM_2.5_ concentration time series for Kallio Station (solid lines—actual values; dashed lines—predicted values).

**Fig 6:**
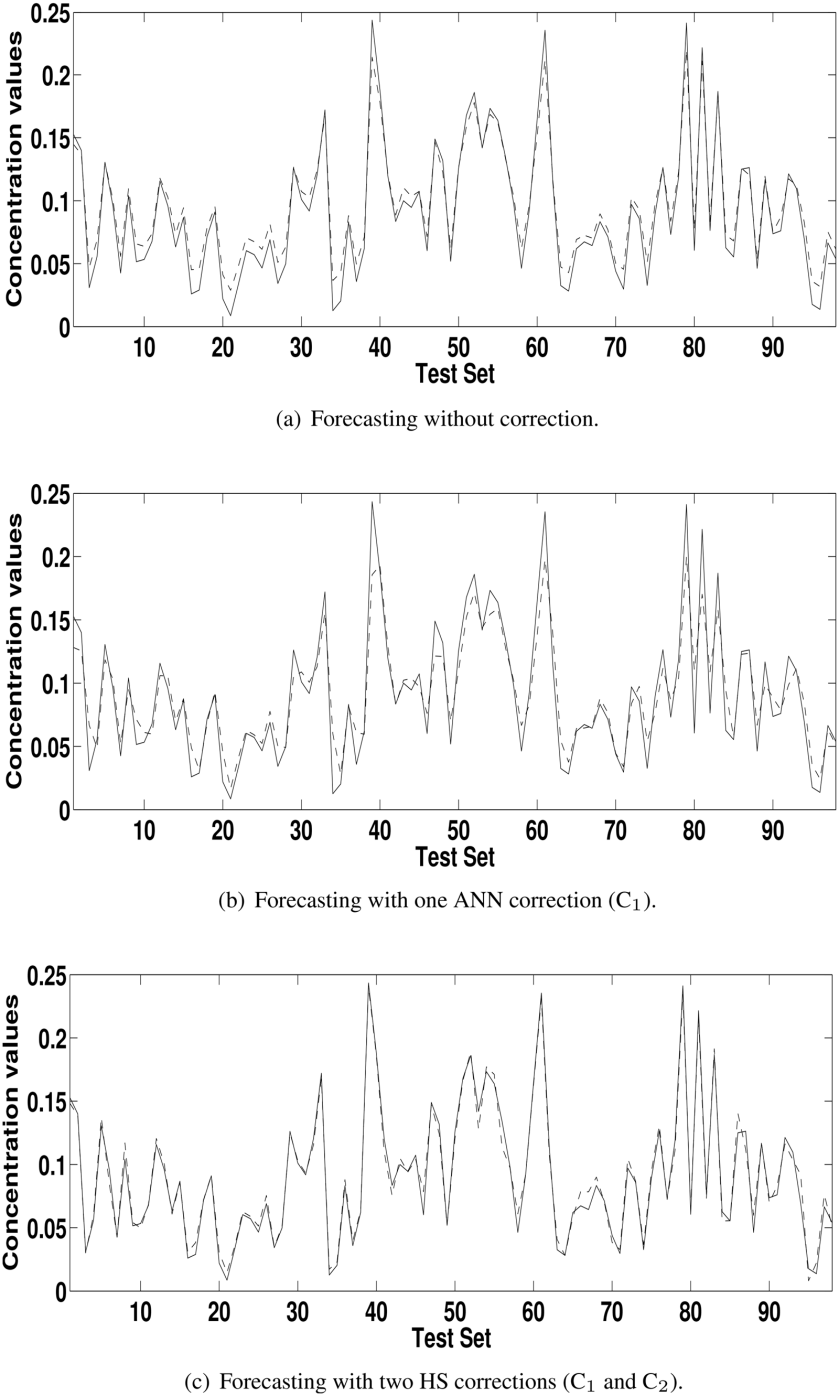
Forecasting for the PM_10_ concentration time series for Vallila Station (solid lines—actual values; dashed lines—predicted values).

**Fig 7:**
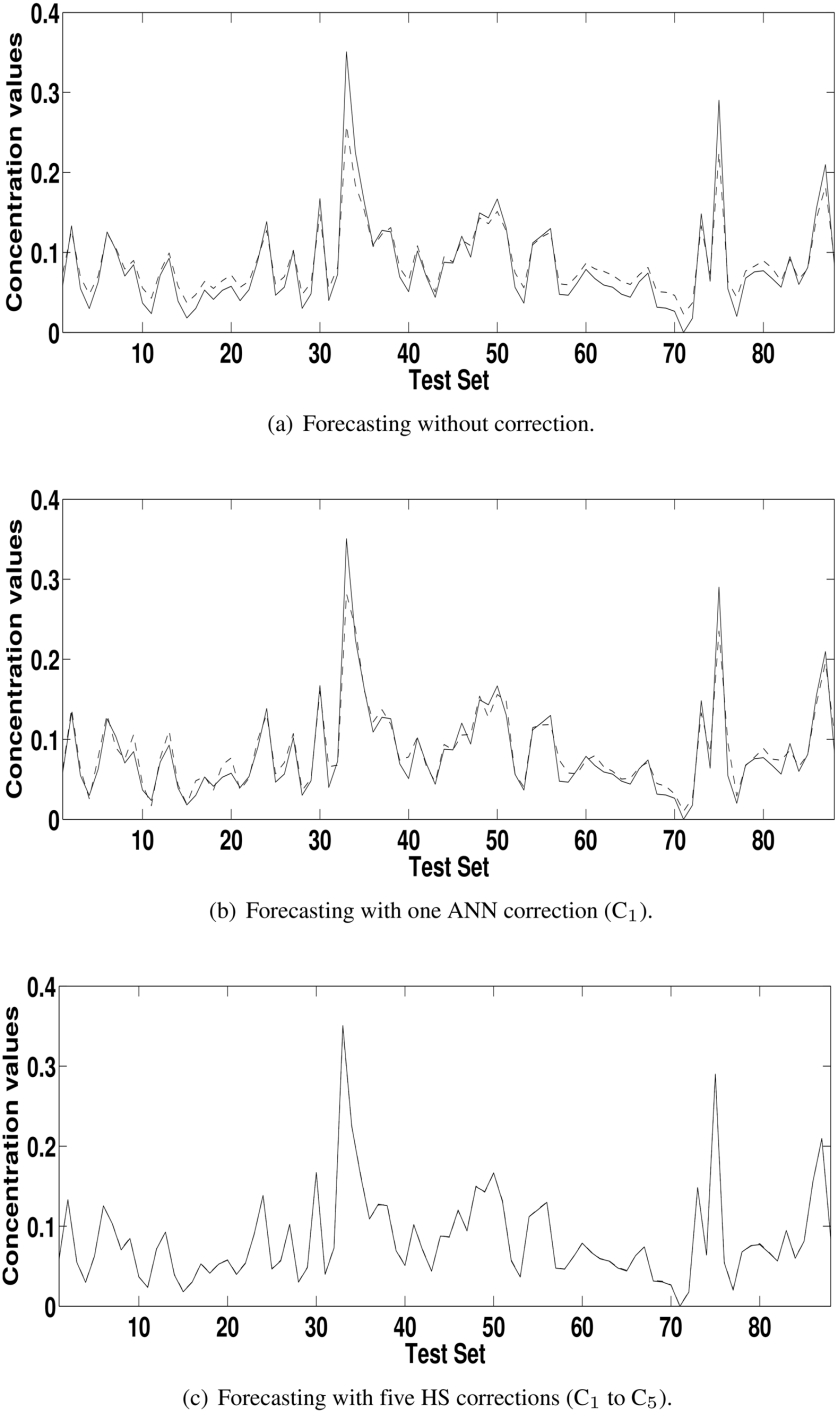
Forecasting for the PM_2.5_ concentration time series for Vallila Station (solid lines—actual values; dashed lines—predicted values).

Table 3 shows the results reached with the model HS+C_1_ and the uncorrected model (HS) according to the ratio defined in the Eq (3). The values greater than 1 for POCID, IA and fitness show that the addition of the correction improved the forecasting of the HS. For MSE, U, MAPE and ARV the logic of the Eq (3) is inverted; values smaller than 1 show forecasting enhancement. For concentration series from Kallio station, greater improvements occurred in U, MAPE and ARV metrics. For concentration series from Vallila station, greater improvement occurred in MAPE metric. In general, the addition of corrections made the forecasting closer to the actual time series. This improvement can be seen by comparing Figs 4(a), 5(a), 6(a) and 7(a) with Figs 4(b), 5(b), 6(b) and 7(b), which are the forecasting for PM_2.5_ and PM_10_ concentration time series from Kallio and Vallila stations without and with correction, respectively.

**Table 3:** Comparison between subsequent corrections with ANN and regarding the model without correction measured for Eq (3) for all series.

Series	Models	MSE	POCID	U	MAPE	ARV	IA	Fitness
**Kallio—PM**_10_	C_1_ − C_0_	0.964	0.896	0.874	0.874	0.637	1.013	1.023
**Kallio—PM**_2.5_	C_1_ − C_0_	0.983	0.903	0.856	0.700	0.658	1.011	1.271
**Vallila—PM**_10_	C_1_ − C_0_	1.000	0.905	1.080	0.845	0.976	1.004	1.064
**Vallila -PM**_2.5_	C_1_ − C_0_	2.012	0.964	1.438	0.705	1.349	1.010	1.337

### Correction via Hybrid System

Table 4 shows the performance of the model uncorrected (HS) and the proposed methodology using HS+C_*n*_ in terms of the evaluation measures and fitness for PM_2.5_ and PM_10_ concentration time series from Kallio and Vallila stations. In the proposed methodology using correction via HS, the number of corrections *n* ranged from 1 to 5. The stopping condition reached was two increases in MAPE value in followed corrections, for all cases. This result suggests that the capacity of correction depends on the accuracy of the forecasting method used in this step. Table 4 shows that the proposed methodology using correction via HS was able to correct the forecasting of the HS more than once in 3 of 4 time series.

**Table 4:** Results with correction via HS for all series. The best result for each metric and fitness is highlighted in bold.

Station	Series	Models	MSE	POCID	U	MAPE	ARV	IA	Fitness
**Kallio**	PM_10_	HS	6.00E-04	**97.16**	0.0969	35.93	0.3077	0.950	2.60
		HS+C_1_	**1.34E-04**	94.29	**0.0193**	**14.39**	**0.0339**	**0.993**	**6.11**
	PM_2.5_	HS	3.00E-04	**97.19**	0.0596	27.41	0.1349	0.970	3.40
		HS+C_1_	5.36E-05	97.09	0.0097	8.87	0.0135	0.997	9.82
		HS+C_1_+C_2_	**1.74E-05**	97.06	**0.0032**	**5.76**	**0.0041**	**0.999**	**14.34**
**Vallila**	PM_10_	HS	3.00E-04	**97.82**	0.0727	29.66	0.1910	0.960	3.16
		HS+C_1_	9.37E-05	95.19	0.0240	14.99	0.0408	0.991	5.93
		HS+C_1_+C_2_	**4.72E-05**	96.12	**0.0121**	**9.19**	**0.0185**	**0.996**	**9.40**
	PM_2.5_	HS	1.00E-04	98.11	0.0313	21.66	0.0630	0.980	4.31
		HS+C_1_	5.26E-05	97.83	0.0122	12.10	0.0210	0.995	7.45
		HS+C_1_+C_2_	9.74E-06	**98.90**	0.0022	4.98	0.0034	0.999	16.54
		HS+C_1_+…+C_3_	9.08E-07	98.89	2.09E-04	1.60	2.85E-04	0.999	38.06
		HS+C_1_+…+C_4_	6.26E-07	98.88	1.42E-04	1.27	1.96E-04	0.999	43.51
		HS+C_1_+…+C_5_	**4.23E-07**	98.86	**9.67E-05**	**0.85**	**1.33E-04**	**0.999**	**53.31**

**Fig 8:**
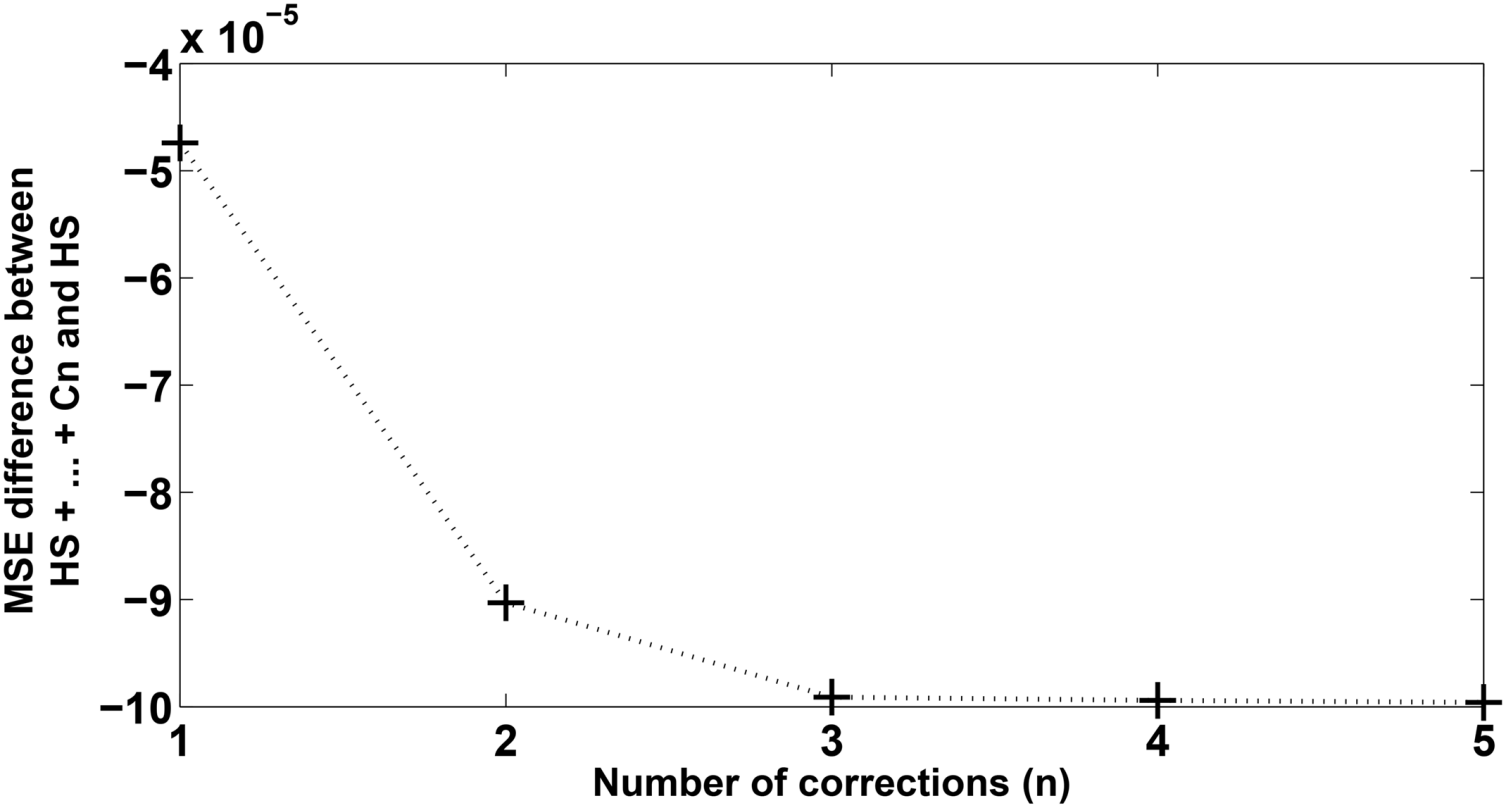
MSE difference between HS with *n* corrections and HS (without correction) for PM_2.5_ concentration series for Vallila station.

Table 4 also shows that the proposed approach improved 21 out of 24 evaluation measures, achieving the best fitness functions for all time series. The best performance for each metric and fitness is highlighted in bold. For pollutant concentration time series from Kallio station, repeating the proposed approach improved almost all evaluation metrics, except the POCID measure. For Vallila station, the improvement also occurred in almost all metrics, except in the POCID measure for PM_10_ concentration time series. For all series, the best model considered was the last model found. For Kallio station, the best models were HS+C_1_ and HS+C_1_+C_2_ for PM_10_ and PM_2.5_, respectively. For Vallila station, the best models were HS+C_1_+C_2_ and HS+C_1_+…+C_5_ for PM_10_ and PM_2.5_, respectively. Table 4 shows that if the values of POCID and fitness are overlooked, the gain in the evaluation measures tends to be smaller at each correction, i.e., at each correction (C_*i*_), the HS aggregated less information than past correction (C_*i*−1_) in the final forecasting. Considering, for instance, Fig 8, it is observed that the MSE difference between HS +…+ Cn and HS (without correction) decreases as the number of corrections increases. The same behavior can be observed in Table 4 in terms of MSE, U, MAPE, ARV and IA, when more than one correction is performed.

Table 5 shows the ratio, according to Eq (3), used to compare (C_*n*_ − C_*n*−1_), the model with *n* corrections with the previous model with *n* − 1 correction terms and the best model with model uncorrected (C_*n*_ − C_0_). As in Table 4, in the Table 5, the values greater than 1 for POCID and IA, and values less than 1 for MSE, U, MAPE and ARV show that the addition of the correction terms improved the forecasting of the HS. The values of fitness greater than 1 at each iteration show that the use of HS in the correction improved the initial forecasting for all time series. The total improvement can be seen in the last lines (C_*n*_ − C_0_) for each time series in Table 5 and by comparing the Figs 4(a), 5(a), 6(a) and 7(a) with Figs 4(c), 5(c), 6(c) and 7(c), which are the forecasting for PM_2.5_ and PM_10_ concentration time series from Kallio and Vallila stations without and with correction, respectively.

**Table 5:** Comparison between subsequent corrections via HS and regarding the model without correction measured for Eq (3) for all series.

Series	Models	MSE	POCID	U	MAPE	ARV	IA	Fitness
**Kallio—PM**_10_	C_1_ − C_0_	0.223	0.970	0.199	0.400	0.110	1.045	2.346
**Kallio—PM**_2.5_	C_1_ − C_0_	0.179	0.999	0.162	0.324	0.100	1.028	2.889
	C_2_ − C_1_	0.324	1.000	0.332	0.650	0.301	1.002	1.461
	C_2_ − C_0_	0.058	0.999	0.054	0.210	0.030	1.030	4.221
**Vallila—PM**_10_	C_1_ − C_0_	0.312	0.973	0.329	0.505	0.213	1.032	1.874
	C_2_ − C_1_	0.504	1.010	0.506	0.613	0.454	1.005	1.586
	C_2_ − C_0_	0.157	0.983	0.167	0.310	0.097	1.037	2.972
**Vallila -PM**_2.5_	C_1_ − C_0_	0.526	0.997	0.390	0.558	0.333	1.016	1.728
	C_2_ − C_1_	0.185	1.011	0.184	0.411	0.160	1.004	2.220
	C_3_ − C_2_	0.093	1.000	0.093	0.321	0.085	1.001	2.302
	C_4_ − C_3_	0.690	1.000	0.682	0.796	0.689	1.000	1.143
	C_5_ − C_4_	0.676	1.000	0.679	0.671	0.678	1.000	1.225
	C_5_ − C_0_	0.004	1.008	0.003	0.039	0.002	1.020	12.365

Comparing the performances of the corrections via ANN and HS from Tables 3 and 5, it can be seen that for all series, the addition of one correction (C_1_ − C_0_) of HS led to better results than ANN. This fact shows that the search for HS through the combination of exploration using gradient descendent algorithms and that using GA, overcomes the use of only one learning algorithm for ANN. Consequently, in cases with correction via HS, where there were more corrections, the proposed methodology improved the performance reached by HS+C_1_.

## Discussion

Table 6 shows for all series, both stations, all the metrics and fitness, the best performance reached via ANN correction and via HS according to Tables 2 and 4, respectively. It is possible to observe, for all cases, that the correction via HS outperforms the correction via ANN. As an example, for PM_10_ of the Vallila station the MSE and ARV results obtained by HS (C_1_+*C*_2_) is one order of magnitude smaller than the one obtained by ANN (C_1_). This result suggests that forecasting methods with high accuracy lead to better corrections. Thus, this study indicates that between the evaluated models, the more indicated for real applications is the HS corrected via HS.

**Table 6:** Comparison between the corrections via ANN and via HS for all series. The best result for each metric and fitness is highlighted in bold.

Station	Series	Correction	MSE	POCID	U	MAPE	ARV	IA	Fitness
**Kallio**	PM_10_	ANN (C_1_)	5.79E-04	87.04	0.0847	31.40	0.1961	0.963	2.66
		HS (C_1_)	**1.34E-04**	**94.29**	**0.0193**	**14.39**	**0.0339**	**0.993**	**5.74**
	PM_2.5_	ANN (C_1_)	2.95E-04	87.74	0.0510	19.18	0.0888	0.981	4.32
		HS (C_1_+C_2_)	**1.74E-05**	**97.06**	**0.0032**	**5.76**	**0.0041**	**0.999**	**14.34**
**Vallila**	PM_10_	ANN (C_1_)	3.00E-04	88.57	0.0785	25.06	0.1864	0.964	3.36
		HS (C_1_+C_2_)	**4.72E-05**	**96.12**	**0.0121**	**9.19**	**0.0185**	**0.996**	**9.40**
	PM_2.5_	ANN (C_1_)	2.01E-04	94.57	0.0450	15.27	0.0850	0.990	5.76
		HS (C_1_+…+C_5_)	**4.23E-07**	**98.86**	**9.67E-05**	**0.85**	**1.33E-04**	**0.999**	**53.31**

**Fig 9:**
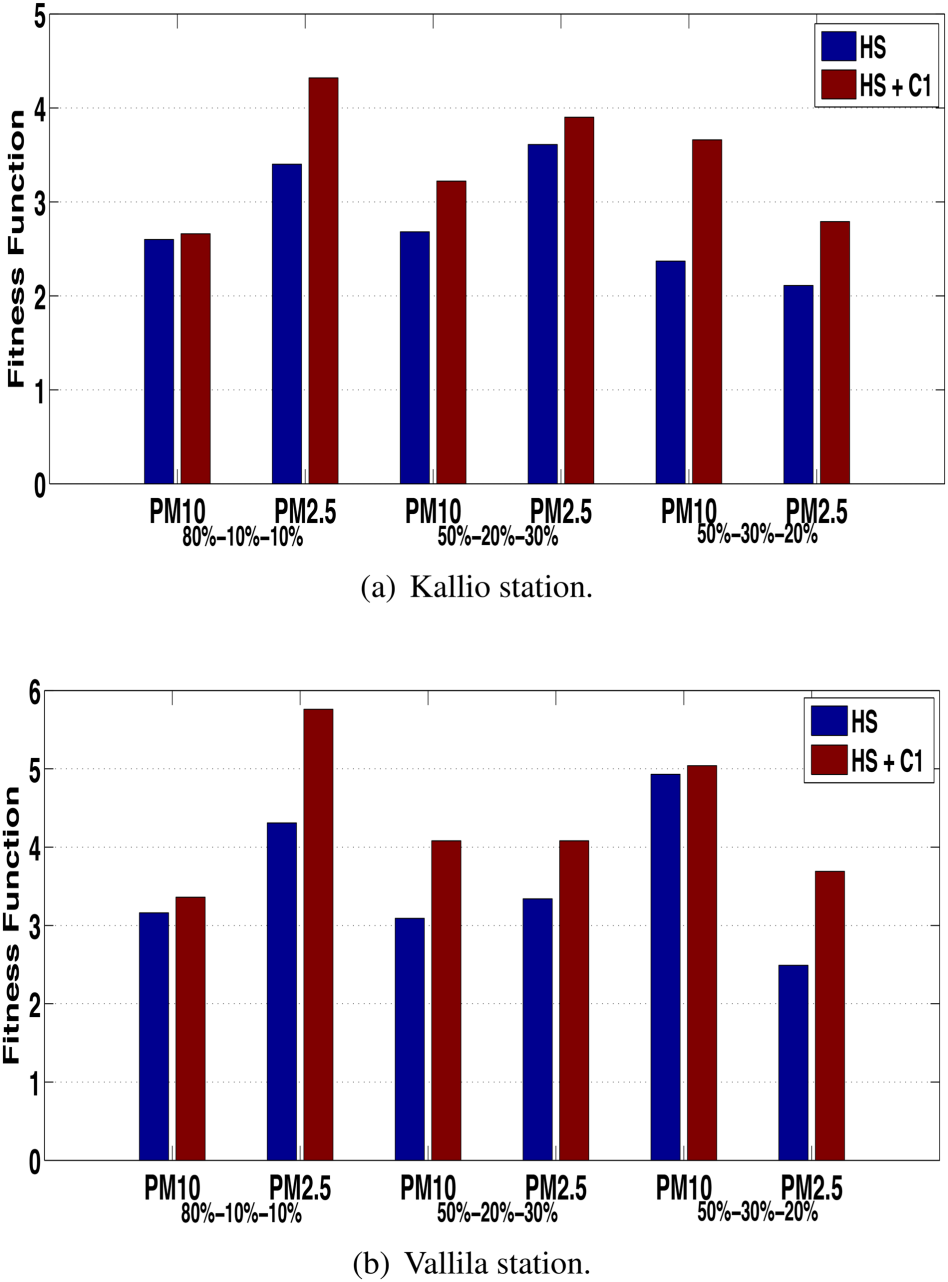
Fitness evolution for Kallio and Vallila stations with correction via ANN.

**Fig 10:**
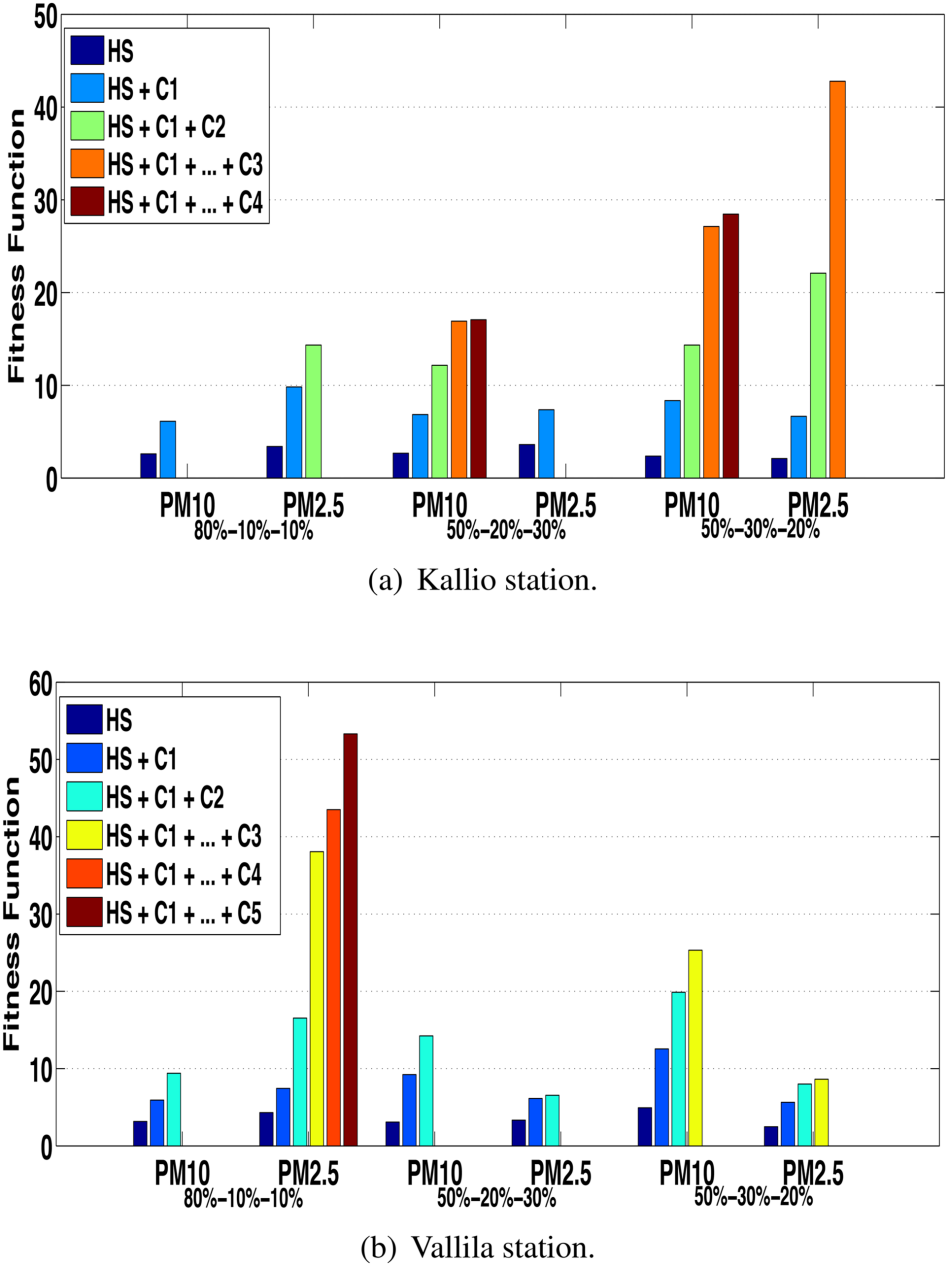
Fitness evolution for Kallio and Vallila stations with correction via HS.

A sensitivity analysis was performed with the objective to show the robustness of the proposed approach in terms of different proportions of the data set. Thus, three proportions are considered for training, validation and testing: 80% − 10% − 10%, 50% − 20% − 30% and 50% − 30% − 20%. Figs 9 and 10 show the results reached for sensitivity analysis in terms of the fitness for all series of both stations for correction via ANN and HS, respectively.

It is observed in Fig 9(a) and 9(b), that when the correction via ANN is performed the fitness value of HS +*C*_1_ is greater than the HS without correction for concentration series in the Kallio and Vallila stations, respectively. For all considered cases, the same stopping criterion of the proposed approach was reached after the first correction (*C*_1_), which was the model residual for a white noise.

Fig 10(a) and 10(b) show the sensitivity analysis of the fitness value with respect to the aforementioned proportions when the correction via HS is considered for Kallio and Vallila stations, respectively. For both stations, the performance at each correction (HS +*C*_*n*_) overcomes the performance of the previous correction (HS +*C*_*n*−1_). For all cases presented in Fig 10 the stopping criterion was the increase in the MAPE value. It is possible to observe that for most cases more than one correction via HS was performed (Fig 10), on the contrary of the correction via ANN, where only one correction was performed in all cases (Fig 9).

**Fig 11:**
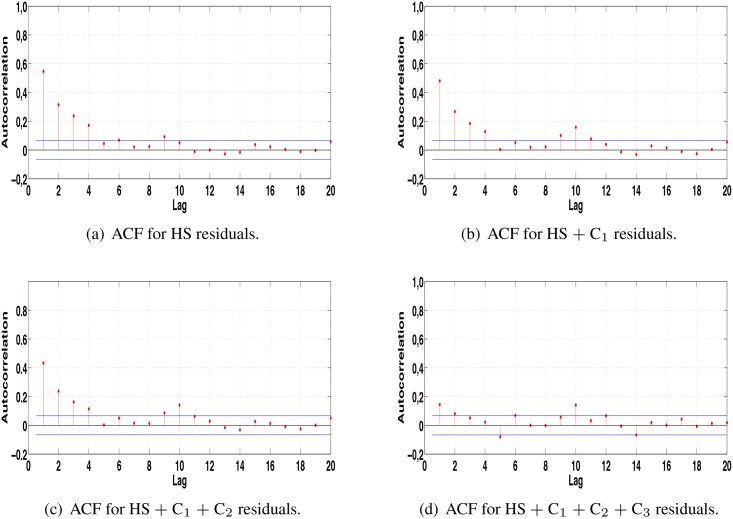
Autocorrelation (ACF) for PM_10_ concentration series for Kallio station.

Fig 11 shows the ACF performed in the residuals obtained after each correction via HS for PM_10_ series with proportion 50% − 30% − 20% for Vallila station (Fig 10(b)). It is possible to observe that none of the residuals, until the correction measured, have white noise behavior. However, Fig 11 shows that at each correction the ACF behavior tends to a white noise. In this case the stopping criteria was the increase in the MAPE value.

## Concluding Remarks

In this paper, a new approach is proposed to improve the performance of particulate matter (PM) forecasting. Important aspects of the proposed approach are highlighted as follows: it uses recursive residuals (error series) modeling; it uses as many residuals as the error series obtained so far is not assumed (by using the ACF) to be a white noise; it is quite general in the sense that it can use a set of different forecasters. Particularly, in the present work, a hybrid system (HS) [19] composed of a GA and ANN is corrected from two methods using residual modeling: an MLP neural network and own HS.

Results were presented in terms of six well-known performance metrics for time series forecasting (mean daily concentration levels) of PM_2.5_ and PM_10_ of two stations from Helsinki: Kallio and Vallila. Results obtained point out the benefits of using recursive residual modeling. An analysis was carried out with the purpose of evaluating the robustness of the proposed approach in terms of different proportions of the data set (training-validation-testing). It was observed that the proposed approach is robust for three different proportions: 80% − 10% − 10%, 50% − 20% − 30% and 50% − 30% − 20%. Thus, as PM concentration can damage human health, the approach presented in this paper may be used as an alternative to PM forecasting, which is a relevant issue to support decisions of organizations and governments for estimating the corresponding health risks.

With objective to investigate the performance of the proposed approach, novel studies will be performed in different scenarios with different combinations of forecasters. Future research directions include: forecasting of other contaminants (e.g. NO, NO_2_, NO_*x*_, CO, O_3_) concentrations; forecasting in the scenario of extreme events (e.g. dust-storm [53, 54]); performance evaluation considering missing data; performance assessment by using series with different times (e.g. second, minute, hour, or month); forecasting with dataset which presents measurement (or observational) errors. Furthermore, a new architecture can be developed with objective to estimate health risks from the forecasting of the proposed approach.
